# *AMELX* Mutations and Genotype–Phenotype Correlation in X-Linked Amelogenesis Imperfecta

**DOI:** 10.3390/ijms25116132

**Published:** 2024-06-01

**Authors:** Shih-Kai Wang, Hong Zhang, Hua-Chieh Lin, Yin-Lin Wang, Shu-Chun Lin, Figen Seymen, Mine Koruyucu, James P. Simmer, Jan C.-C. Hu

**Affiliations:** 1 Department of Dentistry, National Taiwan University School of Dentistry, No. 1, Changde St., Taipei City 100229, Taiwan; lhj830414@ntu.edu.tw (H.-C.L.); wil1019@ntu.edu.tw (Y.-L.W.); r07422010@ntu.edu.tw (S.-C.L.); 2 Department of Pediatric Dentistry, National Taiwan University Children’s Hospital, No. 8, Zhongshan S. Rd., Taipei City 100226, Taiwan; 3 Department of Biologic and Materials Sciences, University of Michigan School of Dentistry, 1011 North University, Ann Arbor, MI 48109, USA; zhanghon@umich.edu (H.Z.); jsimmer@umich.edu (J.P.S.); janhu@umich.edu (J.C.-C.H.); 4 Department of Pediatric Dentistry, Faculty of Dentistry, Altinbas University, Istanbul 34147, Turkey; figen.seymen@altinbas.edu.tr; 5 Department of Pedodontics, Faculty of Dentistry, Istanbul University, Istanbul 34116, Turkey; mine.yildirim@istanbul.edu.tr

**Keywords:** dental enamel, amelogenin, ameloblast, biomineralization, lyonization, signal peptide, protein secretion, ER stress, unfolded protein response, apoptosis

## Abstract

*AMELX* mutations cause X-linked amelogenesis imperfecta (AI), known as AI types IE, IIB, and IIC in Witkop’s classification, characterized by hypoplastic (reduced thickness) and/or hypomaturation (reduced hardness) enamel defects. In this study, we conducted whole exome analyses to unravel the disease-causing mutations for six AI families. Splicing assays, immunoblotting, and quantitative RT-PCR were conducted to investigate the molecular and cellular effects of the mutations. Four *AMELX* pathogenic variants (NM_182680.1:c.2T>C; c.29T>C; c.77del; c.145-1G>A) and a whole gene deletion (NG_012494.2:g.307534_403773del) were identified. The affected individuals exhibited enamel malformations, ranging from thin, poorly mineralized enamel with a “snow-capped” appearance to severe hypoplastic defects with minimal enamel. The c.145-1G>A mutation caused a -1 frameshift (NP_001133.1:p.Val35Cysfs*5). Overexpression of c.2T>C and c.29T>C *AMELX* demonstrated that mutant amelogenin proteins failed to be secreted, causing elevated endoplasmic reticulum stress and potential cell apoptosis. This study reveals a genotype–phenotype relationship for *AMELX*-associated AI: While amorphic mutations, including large deletions and 5′ truncations, of *AMELX* cause hypoplastic-hypomaturation enamel with snow-capped teeth (AI types IIB and IIC) due to a complete loss of gene function, neomorphic variants, including signal peptide defects and 3′ truncations, lead to severe hypoplastic/aplastic enamel (AI type IE) probably caused by “toxic” cellular effects of the mutant proteins.

## 1. Introduction

Amelogenesis imperfecta (AI) is a heterogeneous group of hereditary conditions characterized by developmental enamel defects [1,2]. The prevalence of AI varies between 1 in 700 (northern Sweden) and 1 in 14,000 (USA), depending on the populations studied [3,4]. AI can be inherited in an autosomal-dominant, autosomal-recessive, or X-linked manner. According to Witkop’s classification, which is based upon both the clinical presentations of the enamel malformations and the mode of disease inheritance, AI can be categorized into four main types with 14 subgroups [5]. While AI type I is characterized by changes in enamel thickness (hypoplasia) and AI type II by alterations in enamel hardness (hypomaturation), AI type III is characterized by ultrasoft (hypocalcified) enamel that is susceptible to post-eruptive failure. AI type IV is a special form of enamel malformation with hypoplastic and hypomaturation enamel defects as well as taurodontic root dysmorphology. Among these entries, there are three subtypes showing an X-linked pattern of inheritance: AI types IE, IIB, and IIC (Table 1) [5]. AI type IE describes enamel defects with reduced thickness and smooth surfaces. While affected males have generally thin enamel with brown to yellow-brown discolorations, affected females often show alternating vertical bands of normal and thin enamel due to lyonization [6]. AI type IIB is defined as a hypomaturation type of malformation in which the affected enamel is hypomineralized and shows a reduced radiographic contrast relative to the underlying dentin. Clinically, male patients have soft enamel with a white or mottled yellow ground glass appearance. While the condition was initially characterized as X-linked recessive, a milder disease phenotype can be manifested in heterozygous females due to lyonization. AI type IIC is a specific type of hypomaturation AI described as “snow-capped” teeth. Opaque white enamel covers the incisal or occlusal 1/4 to 1/3 of a tooth crown. The defect can affect all of the primary and permanent teeth, although mandibular incisors are frequently spared [5].

While mutations in many genes have been identified to cause various types of AI, *AMELX* (OMIM *300391) is so far considered as the only responsible gene for X-linked AIs [2,7]. Located at chromosome Xp22.2, human *AMELX* encodes the most abundant enamel matrix protein, amelogenin, secreted by ameloblasts during enamel formation (amelogenesis) [8]. A homologous gene is also present on the Y chromosome, *AMELY* (OMIM *410000), which is transcriptionally active but accounts for only ~10% of the total amelogenin transcripts [9]. Like other genes belonging to the secretory calcium-binding phosphoprotein (SCPP) family, *AMELX* has an untranslated exon 1 and an exon 2 containing the translation initiation site, and codons for the signal peptide and first two amino acids of the secreted protein [10]. All its seven exons are flanked by phase zero introns, which allows alternative RNA splicing without shifting the reading frame. Three transcript variants (TVs) of *AMELX* are listed in the NCBI database. While TV3 (NM_182680.1) includes all the coding exons and encodes the longest isoform 3 (205 a.a.) of amelogenin, TV1 (NM_001142.2) lacks Exon 4 and produces the most abundant AMELX isoform 1 (191 a.a.). Missing Exons 3 and 4, TV2 (NM_182681.1) encodes a shorter isoform 2 (175 a.a.) of the protein [9]. Mouse studies have demonstrated that *Amelx* is exclusively expressed by ameloblasts during the secretory stage of enamel formation. Its expression diminishes rapidly during the post-secretory transition into the maturation stage [11,12,13]. *Amelx* null mice form dysplastic enamel mineral ribbons and produce a thin defective enamel layer, indicating its significant role in sustained elongation rather than initiation of enamel crystals [14]. To date, more than 20 *AMELX* pathogenic variants have been documented to cause different types of X-linked AI [15]. However, how *AMELX* mutations can lead to not only hypoplastic (AI type IE) but hypomaturation (AI types IIB and IIC) enamel defects in humans remains to be elucidated.

Here, we characterized six families with hypoplastic or hypoplastic-hypomaturation AI, and identified four novel and one previously reported pathogenic *AMELX* mutations. Molecular investigation of *AMELX* mutations suggested a probable pathogenic mechanism of ameloblastic cell pathology. Based upon comprehensive analyses of current and reported cases, we propose a genotype–phenotype relationship for *AMELX*-associated AI.

## 2. Results

### 2.1. Family 1 (c.2T>C; p.Met1?)

**Figure 1:**
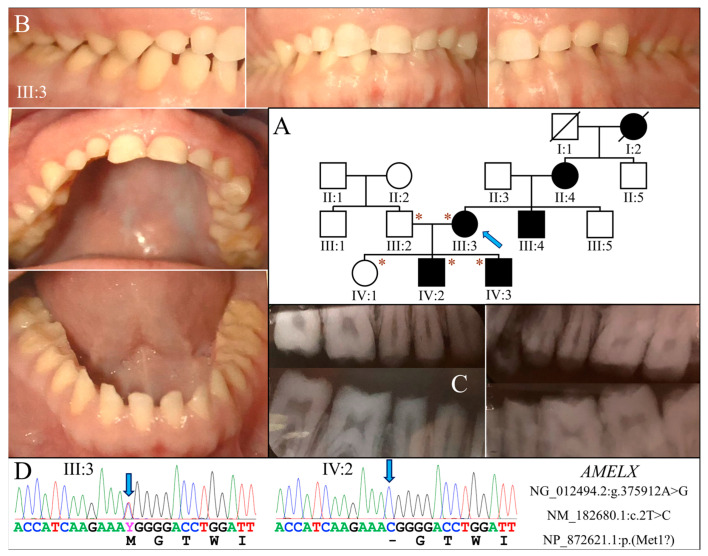
Family 1 with *AMELX* c.2T>C mutation. (**A**) The family pedigree suggests a dominant pattern of disease inheritance. The blue arrow indicates the proband, and the asterisks annotate subjects recruited for genetic testing. (**B**) The proband (III:3) at age 35 had permanent teeth that appeared microdontic and spaced due to severe hypoplastic/aplastic enamel. Tooth surfaces were generally smooth, and dental attrition evident. (**C**) The bitewing radiograph of the proband confirmed the clinical finding of virtually no enamel covering the teeth. (**D**) The DNA sequencing chromatograms identified a single nucleotide transition (g.375912A>G) that abolished the translation initiation codon of *AMELX* (c.2T>C, p.Met1?). While the proband was heterozygous for the mutation, her two sons (IV:2, IV:3) were both hemizygotes.

Family 1 was a Caucasian family in which enamel defects were observed for at least four generations. The proband (III:3) was a 35-year-old female who was otherwise healthy (Figure 1A). Clinically, all her teeth lacked apparent dental enamel, leading to generalized interdental spacing. Chipping of exposed dentin was evident in anterior teeth (Figure 1B). Radiographically, while the dentin layer appeared normal, there was little if any enamel on the tooth (Figure 1C). Her two sons had enamel malformations similar to hers, whereas her husband and daughter were unaffected.

Exome analysis of the proband’s DNA identified a T-to-C transition in *AMELX* (NG_012494.2:g.375912A>G, NM_182680.1:c.2T>C) that changes its translation initiation codon (NP_872621.1:p.Met1?) (Figure 1D). This mutation has previously been reported to cause severe enamel hypoplasia in another AI kindred [16]. Segregation analysis in this family further indicated that both sons were hemizygous for this *AMELX* defect, while the unaffected husband and daughter did not carry the mutation.

### 2.2. Family 2 (c.29T>C, p.Leu10Pro)

**Figure 2:**
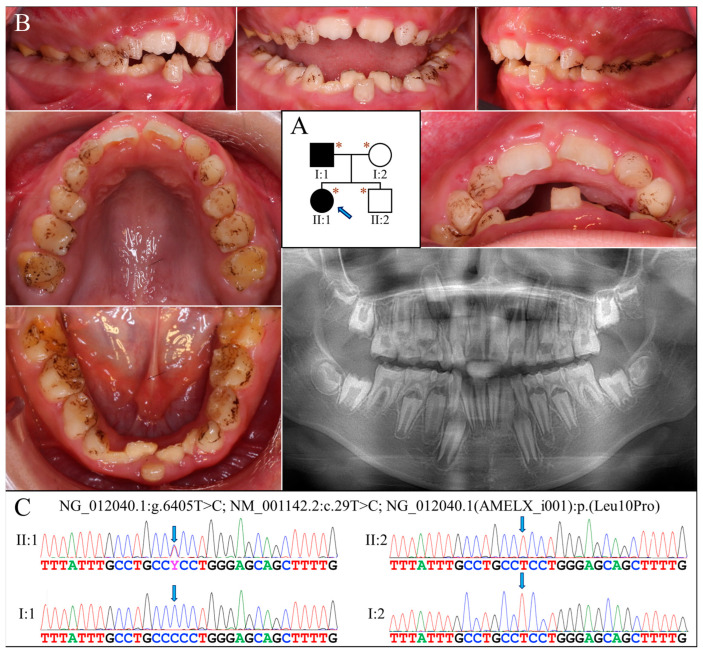
Family 2 with *AMELX* c.29T>C mutation. (**A**) The pedigree shows a nuclear family in which the proband (II:1, age 10) and her father (I:1) were both affected. The blue arrow indicates the proband, and the asterisks annotate subjects recruited for genetic testing. (**B**) The proband had a mixed dentition that exhibited generalized enamel hypoplasia. The surface of the teeth was not particularly rough but presented with black stains. Her maxillary and mandibular dental arches were both markedly narrow. The panoramic radiograph revealed that all her teeth, including unerupted ones, had an extremely thin enamel layer. (**C**) The DNA sequencing chromatograms indicate that the proband and her father were heterozygous and hemizygous, respectively, for the *AMELX* missense mutation (g.375885A>G, c.29T>C, p.Leu10Pro). The unaffected mother (I:2) and younger brother (II:2) did not carry the defect.

Family 2 was a nuclear family in which the proband (II:1, age 10) and her father (I:1) were both affected by hypoplastic AI (Figure 2A). The enamel of both her primary and permanent teeth was extremely thin or absent (Figure 2B). All teeth exhibited rough surface with black stain except for the maxillary permanent central incisors, which were newly erupted. Gingival hyperplasia was not evident. The panoramic radiograph showed a severe enamel hypoplasia or aplasia of all teeth, including developing tooth germs (Figure 2B). No other dental anomalies that resembled enamel renal syndrome (OMIM #204690) were found, such as tooth impaction or pulp calcification. According to the mother, the father had similar enamel defects to those of the proband and has undergone extensive dental treatment for rehabilitation.

Analysis of the proband’s exome revealed no disease-causing mutations in known AI candidate genes except for a novel heterozygous missense mutation in *AMELX* (g.375885A>G, c.29T>C, p.Leu10Pro) (Figure 2C). Although predicted to be benign with a PolyPhen-2 score of 0.003 [17], this mutation substitutes proline for a highly conserved leucine within the signal peptide sequence of AMELX, which might significantly affect its secretion. Also, AlphaMissense, a recently-developed deep learning model that predicts pathogenicity of missense variants and outperforms current algorithms, gives this mutation a pathogenicity score of 0.9161 and predicts it to be likely pathogenic [18]. Further segregation analysis indicated that the proband’s father was hemizygous for this *AMELX* defect, while the proband’s mother and brother both carried wild-type alleles.

### 2.3. Families 3 & 4 (c.77del; p.Pro26Leufs*23)

**Figure 3:**
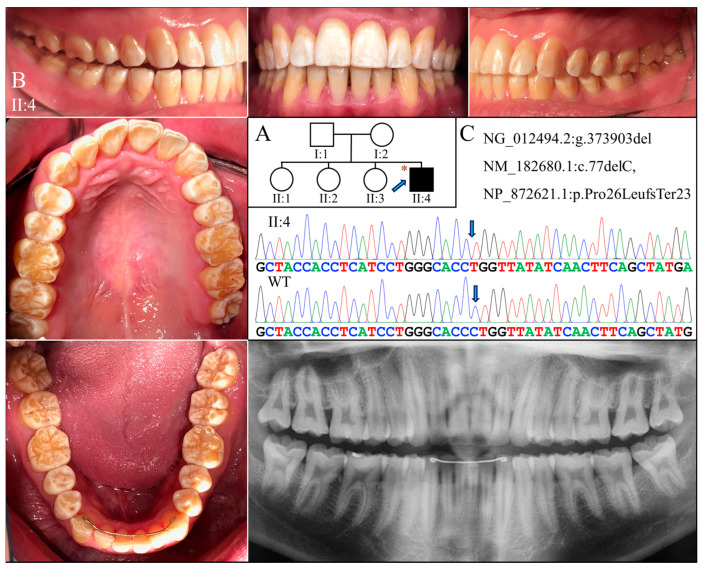
Family 3 with *AMELX* c.77del mutation. (**A**) The pedigree indicates that the proband (II:4) was the only affected individual in the family, although the phenotypes of other members could not be confirmed. The blue arrow indicates the proband, and the asterisks annotate subjects recruited for genetic testing. (**B**) The photographs of the proband at age 20 showed dental crowns with a yellowish-white appearance and lacking normal translucency. The chalky-white discoloration involved more than the incisal or occlusal half of the tooth crowns without a temporal distribution. Dental attrition was evident, particularly over incisal edges and cusp tips. His panorex showed generally thin enamel with reduced radiographic contrast with dentin on all teeth. (**C**) The DNA sequencing chromatogram shows a single nucleotide deletion (g.373903del, c.77del) that caused a frameshift and premature termination (p.Pro26Leufs*23) of the proband’s AMELX protein. The wild-type (WT) chromatogram was generated from an unrelated healthy individual.

The proband of Family 3 (II:4) was a 20-year-old Taiwanese male who was the only individual with AI in the family (Figure 3A). He had a full set of 32 erupted permanent teeth with thin enamel that transmitted the yellowish color of the underlying dentin (Figure 3B). Covering the coronal half of the tooth crown, the hypoplastic enamel appeared chalky white without normal translucency or glaze, indicating a concomitant hypomineralized defect. Dental attrition was evident, particularly over the incisal edges and functional cusps. His panoramic radiograph confirmed the clinical diagnosis of hypoplastic-hypomaturation AI, as a generalized thin enamel layer with reduced radiopacity was observed (Figure 3B).

**Figure 4:**
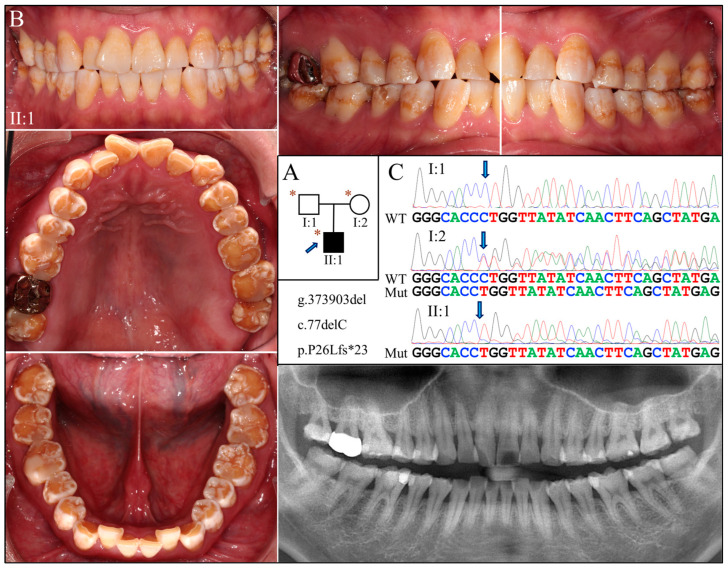
Family 4 with *AMELX* c.77del mutation. (**A**) The pedigree shows a nuclear family in which the proband (II:1) was affected by enamel defects but was otherwise healthy. The blue arrow indicates the proband, and the asterisks annotate subjects recruited for genetic testing. (**B**) The photographs of the proband revealed a dental phenotype of snow-capped teeth similar to that of the Family 3 proband but with more severity. Consistently, the panoramic radiograph indicated a combined hypoplastic and hypomaturation malformation. (**C**) The DNA sequencing chromatogram exhibits the same single nucleotide deletion (g.373903del, c.77del) found in Family 3. While the father (I:1) carried the wild-type *AMELX* allele, the proband (II:1) and the mother (I:2) were hemizygous and heterozygous for the mutation, respectively.

Family 4 was another Taiwanese family unrelated to Family 3 (Figure 4A). The proband was a 27-year-old male who had enamel defects similar to that of the Family 3 proband, although the tooth surface was rougher and dental attrition more pronounced (Figure 4B). His right maxillary second molar was restored with a metal crown due to excessive tooth wear and potential exposure of dental pulp. The radiographic examination findings align with the clinical presentation, indicative of hypoplastic-hypomaturation AI.

Exome analyses for both probands revealed an identical novel single-nucleotide deletion in Exon 3 of *AMELX* (g.373903del, c.77del) (Figure 3C and Figure 4C). The sequence variant, not documented in any of the genome databases we examined, shifts the reading frame and introduces a premature translation termination codon (p.Pro26Leufs*23). The mutation likely results in an amorphic *AMELX* allele, as the mutant transcript will presumably undergo nonsense mediated decay (NMD). According to the proband of Family 3, none of his parents or siblings had tooth defects similar to his, although this could not be validated, as no other family members were available to join the study. In Family 4, while the mother (I:2) was heterozygous for the deletion, the proband (II:1) and the father (I:1) were hemizygous for the mutant and wild-type *AMELX* alleles, respectively (Figure 4C).

### 2.4. Family 5 (c.145-1G>A)

**Figure 5:**
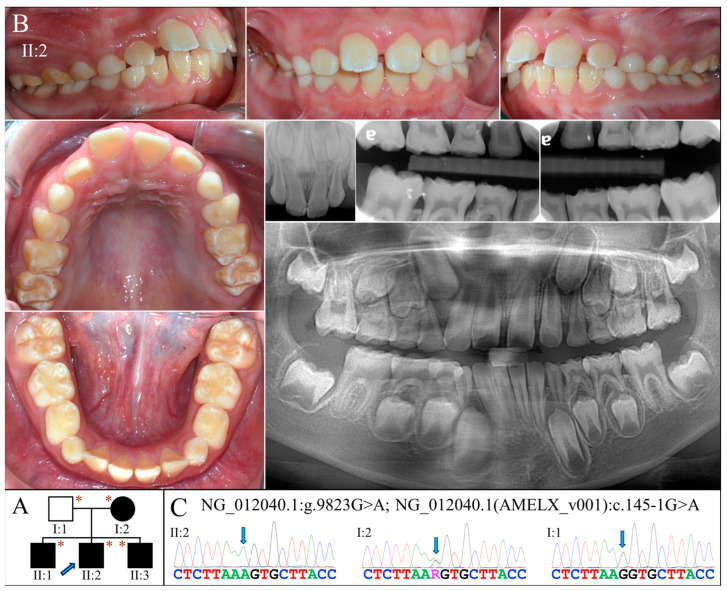
Family 5 with *AMELX* c.145-1G>A mutation. (**A**) The pedigree shows a nuclear family in which all the children were affected and inherited enamel malformations from the mother (I:2). The blue arrow indicates the proband, and the asterisks annotate subjects recruited for genetic testing. (**B**) The proband (II:2, age 10) was at the mixed dentition stage and had enamel defects similar to those of the Family 3 proband. Clinically, the teeth were yellow-white discolored, which resembled snow-capped teeth and suggested a thin and hypomineralized enamel layer. The radiographs confirmed that the enamel was both hypoplastic and hypomature. (**C**) The DNA sequencing chromatogram from the mother showed a G-to-A transition at the splice acceptor site of Intron 4 (g.372467C>T, c.145-1G>A) in one of her *AMELX* genes. While all the (male) children were hemizygous for this variant, the father (I:1) was not.

The proband of Family 5 (II:2) was a 10-year-old boy who was generally healthy (Figure 5A). Clinically, he had an early mixed dentition that showed normal tooth morphology and size. However, the teeth appeared to have thin enamel and displayed the yellowish tone of the underlying dentin (Figure 5B). Some enamel with a chalky-white appearance could be found on the labial surface and incisal edges of the maxillary incisors, and the cusps and marginal ridges of posterior teeth. Radiographically, all the teeth exhibited hypoplastic enamel with a slightly reduced contrast with dentin, including developing tooth germs (Figure 5B). Like the proband, his older (II:1) and younger (II:3) brothers both had similar enamel defects (Figure S1A,B). While the mother had generally normal-looking teeth, sporadic enamel opaque white spots were evident on the facial surfaces of most teeth (Figure S1C). Moderate dental attrition was also noticeable, particularly on the posterior teeth. Radiographically, all her teeth showed enamel of normal thickness and radiopacity. The father was reported to have no apparent dental anomalies, although he was not available for clinical examination.

Whole exome analysis for the proband identified a G-to-A transition at the end of Intron 4 of *AMELX* (g.372467C>T, c.145-1G>A) (Figure 5C). This sequencing variant has never previously been documented or reported. No potential pathogenic mutations were found in other AI candidate genes. The mutation alters the highly conserved splice acceptor site of Intron 4 and presumably alters the splicing of *AMELX* transcripts. Sanger sequencing confirmed that all three affected boys were hemizygotes of this splice-site mutation, while the unaffected father was not. The mother, who showed mild enamel defects, was heterozygous for the variant.

### 2.5. Family 6 (96240 bp Deletion)

**Figure 6:**
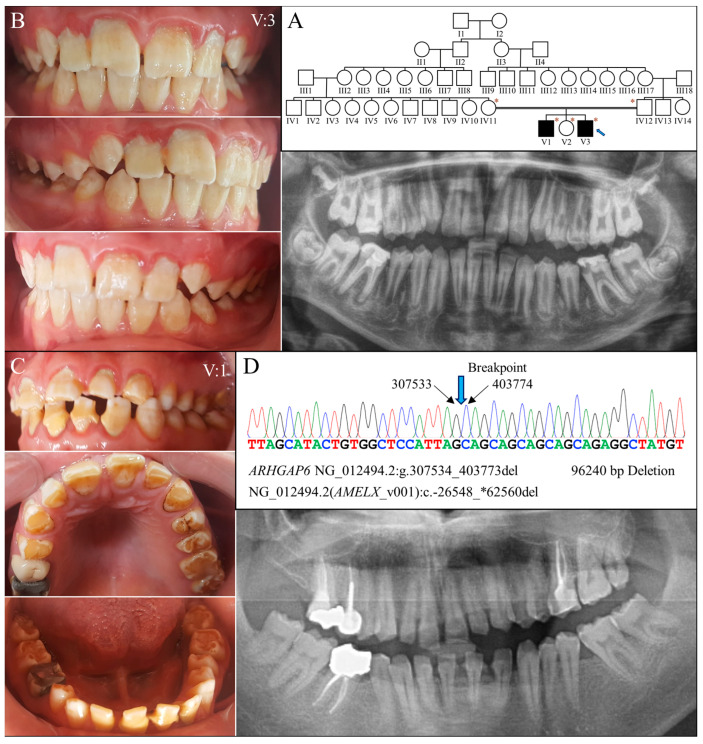
Family 6 with a 96240-bp deletion involving *AMELX*. (**A**) The family pedigree exhibits a consanguineous family in with the two affected individuals were from a second-cousin marriage. The blue arrow indicates the proband, and the asterisks annotate subjects recruited for genetic testing. (**B**) The proband’s (V:3, age 14) teeth appeared rough and chalky white with a yellowish hue. Enamel chipping was evident on maxillary central incisors. Radiographically, the enamel was generally thin and of reduced radiopacity. (**C**) The proband’s brother (V:1, age 20) had a similar enamel phenotype to that of the proband, constituting hypoplastic and hypomaturation AI. (**D**) The DNA sequencing chromatogram from the proband shows a 96240-bp deletion within the Intron 1 of *ARHGAP6* (NG_012494.2:g.307534_403773del) that removes the whole *AMELX* gene, NG_012494.2 (NM_182681.1):c.-26548_*62560del. While the two affected males both carried this deletion and had no *AMELX* gene, their mother (IV:12) was heterozygous to the mutation and had no overt enamel defects.

Family 6 was a large consanguineous family from Turkey. The proband (V:3) and his brother (V:1) were reported to be the only two individuals with AI in the whole family (Figure 6A). At age 14, the proband had all his permanent teeth erupted except the third molars. His teeth exhibited hypoplastic enamel that was chalky white and rough-surfaced (Figure 6B). Tooth fracture or chipping was noted over the maxillary central incisors. Radiographically, all the teeth exhibited a thin enamel layer with reduced contrast relative to the underlying dentin, indicating hypoplastic-hypomaturation AI. At age 20, his older brother had enamel defects similar to his, although the dental attrition and decay were more severe (Figure 6C).

Genomic analysis via whole exome sequencing demonstrated that the proband was hemizygous for a 96,240 base pair deletion at Xp22.2 (g.307534_403773del) (Figure 6D). The defect has never been reported previously and is confined to Intron 1 of *ARHGAP6* (OMIM *300118)- but delete the entire amelogenin gene-, leading to a null *AMELX* allele. Further segregation analysis with Sanger sequencing indicated that the mutation was maternally inherited, as the mother was heterozygous for this defect. The proband’s affected brother (V:1) was hemizygous for the deletion, while his unaffected father (IV:11) and sister (V:2) did not carry this *AMELX* defect.

### 2.6. Molecular Characterization of AMELX Mutations

**Figure 7:**
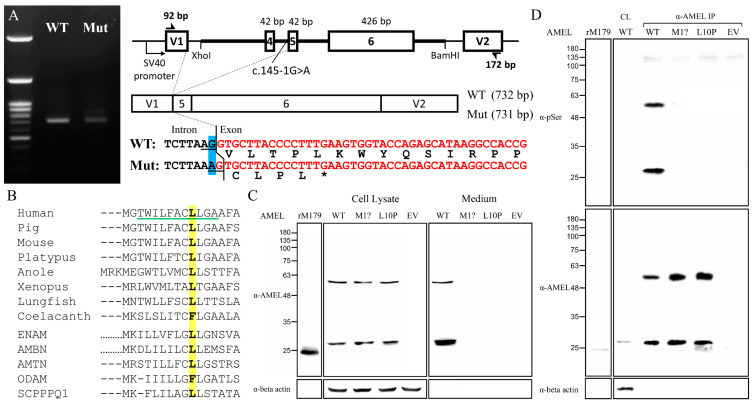
Molecular characterization of *AMELX* mutations. (**A**) Agarose gel electrophoresis exhibited nearly comparable RT-PCR amplicons of 732 and 731 bps from the wild type (WT) and the c.145-1G>A (Mut) minigenes, respectively. The mutant transcript used a new splice acceptor site one nucleotide downstream of the original one, resulting in loss of the first nucleotide of Exon 5 during splicing, which shifted the reading frame and generated a stop codon within the same exon. (**B**) Alignment of the amino acid sequence of human AMELX (NP_001133.1) signal peptide with those of its vertebral orthologs from pig (NP_999071.1), mouse (NP_001075447.1), platypus (XP_001515115.2), anole (XP_003228746.1), Xenopus (NP_001107153.1), lungfish (XP_043928489.1), and coelacanth (XP_005998289.1) as well as other human P/Q-rich SCPPs, including ENAM (NP_114095.2), AMBN (NP_057603.1), AMTN (NP_997722.1), ODAM (NP_060325.3), and SCPPPQ1 (NP_001392157.1). The Leu10 is in bold and highlighted in yellow. The green underline indicates the h-region of the signal peptide. (**C**) Immunoblotting revealed that the mutant amelogenin proteins, p.Met1? and p.Leu10Pro, could only be detected in cell lysates, while the wild-type AMELX was mainly found in the culture medium. (**D**) Anti-phosphoserine (α-pSer) immunoblotting of immunoprecipitated AMELXs only showed positive signals in the wild type. Key: WT, wild type; p.M1?, p.Met1?; p.L10P, p.Leu10Pro; EV, empty vector; rM179, recombinant mouse amelogenin protein; IP, immunoprecipitation.

To investigate how *AMELX* c.145-1G>A splice junction mutation in Family 5 affects mRNA splicing, we conducted minigene splicing assays using pSPL3 constructs that contained a DNA fragment spanning from 3′ region of Intron 3 to 5′ of Intron 6 of human *AMELX* (Figure 7A). Both the wild-type and mutant minigenes gave rise to an expected PCR amplification product of ~730 bps, resembling the most abundant *AMELX* transcript, NM_001142.2, which excludes Exon 4 [9]. However, further sequence analysis of the amplicons demonstrated that the mutant product (731 bps) was 1-bp shorter than the wild type (732 bps) and lacked the first nucleotide of Exon 5. Because this nucleotide is a G, substituting the G of its -1 position with an A created a new (cryptic) splice acceptor, AG. Therefore, the NM_001142.2:c.103-1G>A (NM_182680.1:c.145-1G>A) transition shifted the splice site by one nucleotide at the Intron 4-Exon 5 junction and caused a -1 frameshift (NP_001133.1:p.Val35Cysfs*5). The frameshift would in turn introduce a premature stop codon that likely caused NMD of the mutant transcript.

To evaluate the pathogenicity of the p.Leu10Pro missense mutation in the signal peptide, we first analyzed the signal peptide sequences of amelogenin proteins of selected vertebrates as well as those of other human P/Q-rich SCPP proteins, including ENAM, AMBN, AMTN, ODAM, and SCPPPQ1 [19]. The alignment showed a general sequence homology among the analyzed signal peptides (Figure 7B). The substituted Leu10 is well conserved except for coelacanth AMEL and human ODAM, suggesting that it has functional significance. We further conducted secretion assays to investigate the impact of this signal peptide mutation on protein secretion. The c.2T>C (p.Met1?) defect was also investigated to assess its molecular consequences. Immunoblotting revealed that overexpressed human AMELX proteins from all three constructs gave rise to two bands of ~28 kD and ~56 kD, compared to that of ~24 kD from recombinant mouse amelogenin protein (rM179) (Figure 7C). The higher band presumably resulted from AMELX homodimers. However, while the wild-type AMELX was mostly detected in the culture medium, the signals of p.Met1? and p.Leu10Pro could be found only in the cell lysate, indicating that these two mutant proteins were not properly secreted. The positive cell lysate signal from the unsecreted p.Met1? group indicated that an in-frame translation initiation codon, most likely Met17, was used after Met1 was abolished, which demonstrated that the c.2T>C mutation would produce wild-type amelogenin protein without its signal peptide (p.Met1_Ala16del). To verify that the mutant proteins were not targeted to the secretory pathway, we further evaluated if they were properly phosphorylated. Human amelogenin has only one phosphoserine, Ser32, that requires Golgi casein kinase (FAM20C) in the secretory pathway for its phosphorylation [20,21]. Anti-phosphoserine immunoblotting of purified AMELXs demonstrated that only the wild type, but not p.Met1? and p.Leu10Pro mutant amelogenins, had a phosphoserine, which indicated that the mutant proteins were not targeted to the secretory pathway and likely resided in the cytosol (Figure 7D).

**Figure 8:**
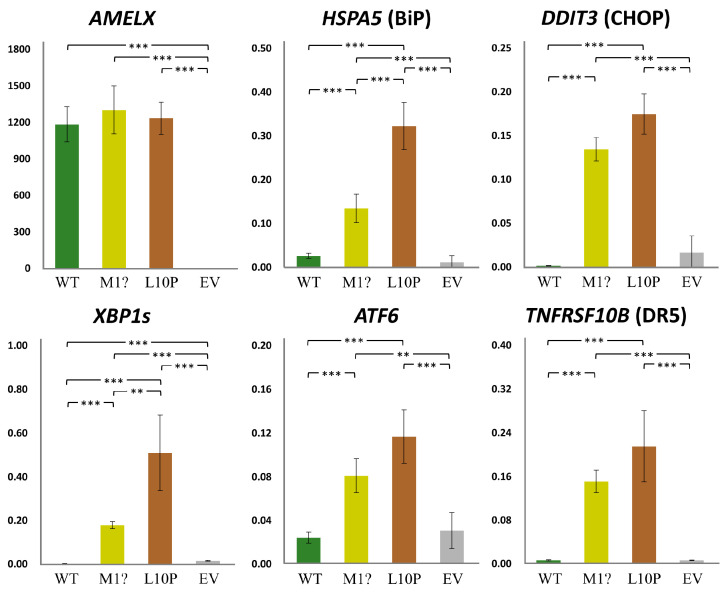
Gene expression under *AMELX* overexpression. Quantitative reverse transcription PCRs (qRT-PCRs) were performed for *AMELX* and ER stress related genes, including *HSPA5*, *DDIT3*, *XBP1s*, and *ATF6*. Expression of *TNFRSF10B*, an apoptotic gene activated by unmitigated ER stress, was also assessed. Key: WT, wild type; p.M1?, p.Met1?; p.L10P, p.Leu10Pro; EV, empty vector; **, *p*-value < 0.05; ***, *p*-value < 0.01.

We further assessed if the mutant non-secreted AMELXs could cause ER stress (ERS) and subsequent cell apoptosis [22]. The qRT-PCRs indicated that while the expression level of overexpressed *AMELX* was comparable among groups, those of ERS-related genes, including *HSPA5*, *DDIT3*, *ATF6*, and spliced *XBP1*, significantly increased in both mutant groups compared to the wild type and the empty vector controls (Figure 8). Furthermore, *TNFRSF10B*, a pro-apoptotic gene induced by unmitigated ERS, was also upregulated when mutant AMELXs were overexpressed. These results demonstrated that the p.Met1? and p.Leu10Pro amelogenin proteins can induce significant ERS (unfolded protein response) and potentially trigger cell apoptosis.

## 3. Discussion

In this study, we report four novel *AMELX* defects, which brings the total number of disease-causing mutations to 30, including four large deletions (Table S1). As these deletions create complete null alleles of *AMELX*, characterizing enamel phenotypes of hemizygous males carrying the mutation provides a great opportunity to investigate the functions of *AMELX* in human amelogenesis. It was previously shown that the affected males from two families with respective 96240-bp (NG_012494.2:g.302534_398773del) and 52654-bp (g.363924_416576del) deletions had generally thin, chalky-white hypomineralized enamel covering the incisal edges, marginal ridges, and cusp tips, giving their teeth a “snow-capped” appearance [23]. A partial *AMELX* deletion of 4723 (g.375892_375900del) bps from Intron 2 to Exon 7, which only leaves the first 18 codons encoding the signal peptide and first two amino acids of the AMELX protein, was reported to cause “hypomaturation AI” in males, although a hypoplastic component of the defects was evident by reviewing the documented dental records [24]. Consistently, our Family 6 proband and his affected brother with the hemizygous g.307534_403773del mutation both had aberrantly thin and chalky-white enamel. Interestingly, all the affected males in Families 3, 4, and 5 also showed a similar phenotype of hypoplastic-hypomaturation AI with a “snow-capped” appearance. Like the c.77del mutation of Families 3 and 4 causing the p.(Pro26Leufs*23) frameshift, our minigene assay demonstrated that the c.145-1G>A variant of Family 5 would lead to a -1 frameshift, p.(Val49Cysfs*5). Both frameshifts will create a premature translation termination codon that triggers transcript degradation by NMD and no expression of mutant *AMELX*. These findings suggest that amorphic mutations equivalent to an *AMELX* null allele cause hypoplastic-hypomaturation enamel with frequent presentation of “snow-capped” teeth in males, designated as AI types IIB and IIC in Witkop’s classification [5]. Scrutinizing the enamel defects of reported male cases with more 5′ truncation mutations (which are most likely to induce NMD), including p.(Pro52Leufs*2) and p.(Pro62Argfs*47), further supports this hypothesis [25,26,27,28]. No complete lack of dental enamel in these *AMELX* null hemizygotes suggested that amelogenin is essential for the appositional growth rather than the initiation of enamel formation. Although it is possible that *AMELY* might compensate for the function of *AMELX*, it has been demonstrated that the *AMELY* transcripts contribute to only ~10% of the total transcripts [9]. Also, both homozygous female and hemizygous male *Amelx* null mice, which have no copy on the Y chromosome [8], were shown to have a thin layer of dental enamel, further backing this conclusion [14,29]. Moreover, the predominant mineral of this thin enamel layer was octacalcium phosphate rather than calcium hydroxyapatite, which might explain the hypomaturation defect of the remaining enamel (snow-capped teeth) in the male patients.

In contrast to the hypoplastic-hypomaturation AI, the enamel malformations of Families 1 and 2, carrying the c.2T>C and c.29T>C variants, respectively, are primarily hypoplastic defects with markedly thin enamel. The c.2T>C mutation has been previously reported in another family with X-linked hypoplastic AI [16]. Theoretically, the variant will abolish the start codon (ATG) and prevent translation initiation. However, it has been suspected that the second ATG, encoding Met17, would be used as an alternative start codon, as it complies with the Kozak consensus sequence for optimal translation initiation, having a purine at the -3 position [30,31]. This would produce a full-length amelogenin protein without the signal peptide, which comprises the first 16 amino acids. Our immunoblotting demonstrated that the protein product of the overexpressed c.2T>C clone could be recognized by an anti-amelogenin antibody and had a comparable molecular weight with the wild-type AMELX, which confirms this hypothesis. However, as expected, the mutant protein, p.Met1_Ala16del, without a signal peptide could not be secreted and would presumably reside in the cytoplasm. Similarly, the amelogenin protein produced by the other mutation, c.29T>C, causing a missense variant, p.Leu10Pro, within the signal peptide was also not properly secreted. In general, a signal peptide contains three structural elements: n-region (N-terminal), h-region (hydrophobic core), and c-region (C-terminal) [32,33]. The h-region, which usually forms an α-helix confirmation, plays a critical role in interacting with the signal recognition particle (SRP), which is an essential step for co-translational protein targeting into the lumen of the endoplasmic reticulum and the secretory pathway [34]. Mutations that alter the hydrophobicity of the h-region have been shown to significantly inhibit protein transport and secretion [35,36]. The signal peptide of human amelogenin contains a predicted h-region of 11 amino acids from Thr3 to Ala13 in which Leu10 is highly conserved among AMELX orthologs [37]. Also, it has been demonstrated that introducing proline, a helix breaker, in a eukaryotic signal peptide can greatly disrupt protein translocation and export [38]. Accordingly, we scrutinized all the available amelogenin protein sequences of vertebrates in the NCBI database and found no signal peptides containing a proline residue. Therefore, the p.Leu10Pro substitution probably abrogates the interaction of signal peptide with SRP, prevents protein translocation into the ER, and causes the mutant amelognin to reside in the cytoplasm like the p.Met1_Ala16del AMELX. Presumably, all the other four signal peptide mutations of *AMELX* reported to date would result in a similar effect and inhibit amelogenin secretion [7,16,39].

Noticeably, all these start codon and signal peptide mutations cause severe hypoplastic AI rather than the hypoplastic-hypomaturation or snow-capped defects that were previously discussed, suggesting a pathogenic mechanism other than complete loss of *AMELX* [15]. Our overexpression study indicated that both p.Met1_Ala16del and p.Leu10Pro AMELXs could induce ER stress, trigger UPR, and potentiate cell apoptosis, which might explain the severe hypoplastic enamel defects. Consistently, AI patients carrying the more 3′ truncation mutations of *AMELX*, including p.(His129Thrfs*60), p.(Tyr141Thrfs*48), p.(Pro158Hisfs*31), p.(Pro173Leufs*16), p.(Leu181Cysfs*8), and p.(Glu191*), have also been reported to have drastically hypoplastic enamel [7,27,40,41,42,43]. Like those of *AMELX* signal peptide mutations, the severe defects likely result from ameloblast cell pathology caused by the aberrant amelogenin, as these mutant transcripts can presumably escape NMD and generate truncated proteins. Further investigations, including studies on diseased cells and animal models, are warranted to test this hypothesis.

In summary, this study established a plausible genotype–phenotype correlation in X-linked (*AMELX*-associated) AI. While large deletions and 5′ truncation mutations of *AMELX* cause hypoplastic-hypomaturation AI with frequent snow-capped appearances (AI types IIB and IIC) due to a complete loss of gene function (amorph), the signal peptide variants and 3′ truncations lead to severe hypoplastic defects of dental enamel (AI type IE) probably through “toxic” cellular effects of the unsecreted mutant protein (neomorph). Conceivably, these phenotypes are readily observed in hemizygous male patients but can be highly variable in heterozygous females due to lyonization.

## 4. Materials and Methods

### 4.1. Genetic Analyses of AI Families

The human research protocols and consent forms were reviewed and approved by the IRB committees at the University of Michigan, University of Istanbul, and the National Taiwan University Hospital. Following comprehensive explanations of the research procedures and addressing any inquiries, the study participants provided their consent by signing written agreements. Oro-dental phenotypes were evaluated through clinical and radiographic examinations, and family pedigrees were constructed via history taking. Unstimulated saliva samples of 2 mL were collected from all available subjects to obtain genomic DNA for mutational analyses. All the procedures were specified in approved study protocols and in compliance with the Declaration of Helsinki.

To discern the AI-causing mutations, whole exome analyses were performed for all available family members of Families 1 and 6 and the probands of Families 2, 3, 4, and 5. Sanger sequencing was conducted to validate detected sequence variants and their segregation with the disease phenotype in each family.

### 4.2. Minigene Splicing Assay

A 1399-bp DNA fragment spanning from Intron 3 to Intron 6 of human *AMELX* (NG_012494.2:g.371542_372940) was amplified from the genomic DNA of the Family 5 proband and subcloned into the pSPL3 exon-trapping vector, using XhoI and BamHI restriction enzyme sites, to generate a mutant *AMELX* minigene containing the NM_182680.1:c.145-1G>A splice-site mutation. A wild-type construct was further produced by correcting the mutation via site-directed mutagenesis. The two plasmids were transfected into HEK293T cells, respectively, and the expressed minigene transcripts were amplified, isolated electrophoretically, and sequenced as previously described [44].

### 4.3. Secretion Assay and Quantitative Reverse Transcription PCR (qRT-PCR)

A human *AMELX* expression clone was constructed by inserting a partial *AMELX* cDNA transcript (NM_001142.2:c.-10_576) into the pcDNATM3.1(+) vector to overexpress the wild-type isoform 1 of amelogenin protein (NP_001133.1). Two mutant constructs, annotated as p.Met1? and p.Leu10Pro, were further generated by introducing c.2T>C and c.29T>C mutations, respectively. These 3 plasmids along with the empty vector were transfected into HEK293T cells, and the following assays performed 72 h following transfection. For evaluation of protein secretion, AMEL immunoblotting was conducted for the cell lysate and culture media using a mouse monoclonal antibody (F-11) raised against full-length human amelogenin isoform 1 (1:500; sc-365284; Santa Cruz Biotechnology, Dallas, TX, USA) and an anti-beta actin (1:5000; ab8226; Abcam, Cambridge, UK) antibody. To detect phosphoserine in AMELs, overexpressed proteins were first immunoprecipitated from cell lysates using the F-11 antibody and probed with anti-phosphoserine antibody (1:1000; AB1603; MilliporeSigma, Burlington, MA, USA). Real-time reverse transcription PCR (qRT-PCR) was carried out as previously described [45].

## Figures and Tables

**Table 1:** Dental phenotypes of the 3 X-linked AI types in Witkop’s classification.

Witkop’s AI Types	Clinical Presentations	Radiographic Findings
Type IE (hypoplastie, smooth X-linked dominant)	Males: smooth, shiny, and thin enamel with yellow-brown discoloration; aOB ^1^ in most cases Females: alternating vertical hypoplastic bands of enamel; aOB in ~1/3 cases	Males: generally thin enamel coverage of all teeth; less frequent crown resorption of unerupted teeth Females: vertical banding of enamel
Type IIB (hypomaturation, X-linked recessive)	Males: soft enamel with ground-glass white appearance in primary teeth and mottled yellow in permanents Females: alternating vertical bands of translucent normal enamel and discoloration similar to those of affected males	Males: reduced radiographic contrast between enamel and dentin Females: no overt defects
Type IIC (snow-capped teeth)	Opaque white enamel covering the incisal/occlusal 1/4 to 1/3 crown portion of both primary and permanent teeth; no temporal distribution as seen in enamel defects caused by environmental factors, such as fluorosis	-

^1^ aOB: anterior open bite.

## Data Availability

Whole exome sequencing data and analysis as well as additional data used to support the findings of this study are available from the corresponding author upon request.

## Supplementary Materials

The following supporting information can be downloaded at: https://www.mdpi.com/article/10.3390/ijms25116132/s1, References [7,15,16,23,24,25,26,27,28,39,40,41,42,43,46,47,48,49,50,51,52,53,54,55,56,57] are cited in the supplementary materials.

## References

[1] Hu J.C., Chun Y.H., Al Hazzazzi T., Simmer J.P. (2007). Enamel formation and amelogenesis imperfecta. Cells Tissues Organs.

[2] Smith C.E.L., Poulter J.A., Antanaviciute A., Kirkham J., Brookes S.J., Inglehearn C.F., Mighell A.J. (2017). Amelogenesis Imperfecta; Genes, Proteins, and Pathways. Front. Physiol..

[3] Bäckman B., Holm A.K. (1986). Amelogenesis imperfecta: Prevalence and incidence in a northern Swedish county. Community Dent. Oral. Epidemiol..

[4] Witkop C.J. (1957). Hereditary defects in enamel and dentin. Acta Genet. Stat. Med..

[5] Witkop C.J. (1988). Amelogenesis imperfecta, dentinogenesis imperfecta and dentin dysplasia revisited: Problems in classification. J. Oral. Pathol..

[6] Lyon M.F. (2003). The Lyon and the LINE hypothesis. Semin. Cell Dev. Biol..

[7] Bloch-Zupan A., Rey T., Jimenez-Armijo A., Kawczynski M., Kharouf N., Dure-Molla M., Noirrit E., Hernandez M., Joseph-Beaudin C., Lopez S. (2023). Amelogenesis imperfecta: Next-generation sequencing sheds light on Witkop’s classification. Front. Physiol..

[8] Lau E.C., Mohandas T.K., Shapiro L.J., Slavkin H.C., Snead M.L. (1989). Human and mouse amelogenin gene loci are on the sex chromosomes. Genomics.

[9] Salido E.C., Yen P.H., Koprivnikar K., Yu L.C., Shapiro L.J. (1992). The human enamel protein gene amelogenin is expressed from both the X and the Y chromosomes. Am. J. Hum. Genet..

[10] Kawasaki K., Weiss K.M. (2006). Evolutionary genetics of vertebrate tissue mineralization: The origin and evolution of the secretory calcium-binding phosphoprotein family. J. Exp. Zool. B Mol. Dev. Evol..

[11] Hu J.C., Sun X., Zhang C., Simmer J.P. (2001). A comparison of enamelin and amelogenin expression in developing mouse molars. Eur. J. Oral. Sci..

[12] Wurtz T., Lundmark C., Christersson C., Bawden J.W., Slaby I., Hammarström L. (1996). Expression of amelogenin mRNA sequences during development of rat molars. J. Bone Miner. Res..

[13] Wakida K., Amizuka N., Murakami C., Satoda T., Fukae M., Simmer J.P., Ozawa H., Uchida T. (1999). Maturation ameloblasts of the porcine tooth germ do not express amelogenin. Histochem. Cell Biol..

[14] Hu Y., Smith C.E., Cai Z., Donnelly L.A., Yang J., Hu J.C., Simmer J.P. (2016). Enamel ribbons, surface nodules, and octacalcium phosphate in C57BL/6 Amelx(-/-) mice and Amelx(+/-) lyonization. Mol. Genet. Genomic Med..

[15] Leban T., Trebušak Podkrajšek K., Kovač J., Fidler A., Pavlič A. (2022). An Intron c.103-3T>C Variant of the AMELX Gene Causes Combined Hypomineralized and Hypoplastic Type of Amelogenesis Imperfecta: Case Series and Review of the Literature. Genes.

[16] Kim J.W., Simmer J.P., Hu Y.Y., Lin B.P., Boyd C., Wright J.T., Yamada C.J., Rayes S.K., Feigal R.J., Hu J.C. (2004). Amelogenin p.M1T and p.W4S mutations underlying hypoplastic X-linked amelogenesis imperfecta. J. Dent. Res..

[17] Adzhubei I.A., Schmidt S., Peshkin L., Ramensky V.E., Gerasimova A., Bork P., Kondrashov A.S., Sunyaev S.R. (2010). A method and server for predicting damaging missense mutations. Nat. Methods.

[18] Cheng J., Novati G., Pan J., Bycroft C., Žemgulytė A., Applebaum T., Pritzel A., Wong L.H., Zielinski M., Sargeant T. (2023). Accurate proteome-wide missense variant effect prediction with AlphaMissense. Science.

[19] Kawasaki K., Weiss K.M. (2008). SCPP gene evolution and the dental mineralization continuum. J. Dent. Res..

[20] Fincham A.G., Simmer J.P. (1997). Amelogenin proteins of developing dental enamel. Ciba Found. Symp..

[21] Tagliabracci V.S., Engel J.L., Wen J., Wiley S.E., Worby C.A., Kinch L.N., Xiao J., Grishin N.V., Dixon J.E. (2012). Secreted kinase phosphorylates extracellular proteins that regulate biomineralization. Science.

[22] Walter P., Ron D. (2011). The unfolded protein response: From stress pathway to homeostatic regulation. Science.

[23] Hu J.C., Chan H.C., Simmer S.G., Seymen F., Richardson A.S., Hu Y., Milkovich R.N., Estrella N.M., Yildirim M., Bayram M. (2012). Amelogenesis imperfecta in two families with defined AMELX deletions in ARHGAP6. PLoS ONE.

[24] Lagerström M., Dahl N., Nakahori Y., Nakagome Y., Bäckman B., Landegren U., Pettersson U. (1991). A deletion in the amelogenin gene (AMG) causes X-linked amelogenesis imperfecta (AIH1). Genomics.

[25] Aldred M.J., Crawford P.J., Roberts E., Thomas N.S. (1992). Identification of a nonsense mutation in the amelogenin gene (AMELX) in a family with X-linked amelogenesis imperfecta (AIH1). Hum. Genet..

[26] Lench N.J., Brook A.H., Winter G.B. (1994). SSCP detection of a nonsense mutation in exon 5 of the amelogenin gene (AMGX) causing X-linked amelogenesis imperfecta (AIH1). Hum. Mol. Genet..

[27] Wright J.T., Torain M., Long K., Seow K., Crawford P., Aldred M.J., Hart P.S., Hart T.C. (2011). Amelogenesis imperfecta: Genotype-phenotype studies in 71 families. Cells Tissues Organs.

[28] Duan X., Yang S., Zhang H., Wu J., Zhang Y., Ji D., Tie L., Boerkoel C.F. (2019). A Novel AMELX Mutation, Its Phenotypic Features, and Skewed X Inactivation. J. Dent. Res..

[29] Bartlett J.D., Smith C.E., Hu Y., Ikeda A., Strauss M., Liang T., Hsu Y.H., Trout A.H., McComb D.W., Freeman R.C. (2021). MMP20-generated amelogenin cleavage products prevent formation of fan-shaped enamel malformations. Sci. Rep..

[30] Hernández G., Osnaya V.G., Pérez-Martínez X. (2019). Conservation and Variability of the AUG Initiation Codon Context in Eukaryotes. Trends Biochem. Sci..

[31] Kozak M. (1981). Possible role of flanking nucleotides in recognition of the AUG initiator codon by eukaryotic ribosomes. Nucleic Acids Res..

[32] Martoglio B., Dobberstein B. (1998). Signal sequences: More than just greasy peptides. Trends Cell Biol..

[33] von Heijne G. (1985). Signal sequences. The limits of variation. J. Mol. Biol..

[34] Hatsuzawa K., Tagaya M., Mizushima S. (1997). The hydrophobic region of signal peptides is a determinant for SRP recognition and protein translocation across the ER membrane. J. Biochem..

[35] Nilsson I., Lara P., Hessa T., Johnson A.E., von Heijne G., Karamyshev A.L. (2015). The code for directing proteins for translocation across ER membrane: SRP cotranslationally recognizes specific features of a signal sequence. J. Mol. Biol..

[36] Karamyshev A.L., Patrick A.E., Karamysheva Z.N., Griesemer D.S., Hudson H., Tjon-Kon-Sang S., Nilsson I., Otto H., Liu Q., Rospert S. (2014). Inefficient SRP interaction with a nascent chain triggers a mRNA quality control pathway. Cell.

[37] Teufel F., Almagro Armenteros J.J., Johansen A.R., Gíslason M.H., Pihl S.I., Tsirigos K.D., Winther O., Brunak S., von Heijne G., Nielsen H. (2022). SignalP 6.0 predicts all five types of signal peptides using protein language models. Nat. Biotechnol..

[38] Ryan P., Edwards C.O. (1995). Systematic introduction of proline in a eukaryotic signal sequence suggests asymmetry within the hydrophobic core. J. Biol. Chem..

[39] Lagerström-Fermér M., Nilsson M., Bäckman B., Salido E., Shapiro L., Pettersson U., Landegren U. (1995). Amelogenin signal peptide mutation: Correlation between mutations in the amelogenin gene (AMGX) and manifestations of X-linked amelogenesis imperfecta. Genomics.

[40] Lench N.J., Winter G.B. (1995). Characterisation of molecular defects in X-linked amelogenesis imperfecta (AIH1). Hum. Mutat..

[41] Hart P.S., Aldred M.J., Crawford P.J., Wright N.J., Hart T.C., Wright J.T. (2002). Amelogenesis imperfecta phenotype-genotype correlations with two amelogenin gene mutations. Arch. Oral. Biol..

[42] Lee K.E., Lee S.K., Jung S.E., Song S.J., Cho S.H., Lee Z.H., Kim J.W. (2011). A novel mutation in the AMELX gene and multiple crown resorptions. Eur. J. Oral. Sci..

[43] Kindelan S.A., Brook A.H., Gangemi L., Lench N., Wong F.S., Fearne J., Jackson Z., Foster G., Stringer B.M. (2000). Detection of a novel mutation in X-linked amelogenesis imperfecta. J. Dent. Res..

[44] Chu K.Y., Wang Y.L., Chen J.T., Lin C.H., Yao C.J., Chen Y.J., Chen H.W., Simmer J.P., Hu J.C., Wang S.K. (2023). PAX9 mutations and genetic synergism in familial tooth agenesis. Ann. N. Y. Acad. Sci..

[45] Wang Y.L., Lin H.C., Liang T., Lin J.C., Simmer J.P., Hu J.C., Wang S.K. (2024). ENAM Mutations Can Cause Hypomaturation Amelogenesis Imperfecta. J. Dent. Res..

[46] Cho E.S., Kim K.J., Lee K.E., Lee E.J., Yun C.Y., Lee M.J., Shin T.J., Hyun H.K., Kim Y.J., Lee S.H. (2014). Alteration of conserved alternative splicing in AMELX causes enamel defects. J. Dent. Res..

[47] Kim Y.J., Kang J., Seymen F., Koruyucu M., Zhang H., Kasimoglu Y., Bayram M., Tuna-Ince E.B., Bayrak S., Tuloglu N. (2020). Alteration of Exon Definition Causes Amelogenesis Imperfecta. J. Dent. Res..

[48] Kida M., Sakiyama Y., Matsuda A., Takabayashi S., Ochi H., Sekiguchi H., Minamitake S., Ariga T. (2007). A novel missense mutation (p.P52R) in amelogenin gene causing X-linked amelogenesis imperfecta. J. Dent. Res..

[49] Prasad M.K., Geoffroy V., Vicaire S., Jost B., Dumas M., Le Gras S., Switala M., Gasse B., Laugel-Haushalter V., Paschaki M. (2016). A targeted next-generation sequencing assay for the molecular diagnosis of genetic disorders with orodental involvement. J. Med. Genet..

[50] Chan H.C., Estrella N.M., Milkovich R.N., Kim J.W., Simmer J.P., Hu J.C. (2011). Target gene analyses of 39 amelogenesis imperfecta kindreds. Eur. J. Oral Sci..

[51] Collier P.M., Sauk J.J., Rosenbloom S.J., Yuan Z.A., Gibson C.W. (1997). An amelogenin gene defect associated with human X-linked amelogenesis imperfecta. Arch. Oral Biol..

[52] Hart S., Hart T., Gibson C., Wright J.T. (2000). Mutational analysis of X-linked amelogenesis imperfecta in multiple families. Arch. Oral Biol..

[53] Ravassipour D.B., Hart P.S., Hart T.C., Ritter A.V., Yamauchi M., Gibson C., Wright J.T. (2000). Unique enamel phenotype associated with amelogenin gene (AMELX) codon 41 point mutation. J. Dent. Res..

[54] Kim Y.J., Kim Y.J., Kang J., Shin T.J., Hyun H.K., Lee S.H., Lee Z.H., Kim J.W. (2017). A novel AMELX mutation causes hypoplastic amelogenesis imperfecta. Arch. Oral Biol..

[55] Sekiguchi H., Alaluusua S., Minaguchi K., Yakushiji M. (2001). A new mutation in the amelogenin gene causes X-linked amelogenesis imperfecta. J. Dent. Res..

[56] Greene S.R., Yuan Z.A., Wright J.T., Amjad H., Abrams W.R., Buchanan J.A., Trachtenberg D.I., Gibson C.W. (2002). A new frameshift mutation encoding a truncated amelogenin leads to X-linked amelogenesis imperfecta. Arch. Oral Biol..

[57] Zhang Z., Zou X., Feng L., Huang Y., Chen F., Sun K., Song Y., Lv P., Gao X., Dong Y. (2023). Splicing mutations in AMELX and ENAM cause amelogenesis imperfecta. BMC Oral Health.

