# Supplementary material for: AMELX Mutations and Genotype–Phenotype Correlation in X-Linked Amelogenesis Imperfecta
Source: Int J Mol Sci. 2024 Jun 1;25(11):6132. doi: 10.3390/ijms25116132 (PMC11172428; doi:10.3390/ijms25116132)
Supplement: Supplementary file 1 [file ijms-25-06132-s001.zip › ijms-3003435-supplementary.pdf]

Supplementary Materials

AMELX Mutations and Genotype-Phenotype Correlation in X-linked Amelogenesis Imperfecta

Shih-Kai Wang[1,2*], Hong Zhang[3], Hua-Chieh Lin[1], Yin-Lin Wang[1,2], Shu-Chun Lin[1,2], Figen Seymen[4], Mine Koruyucu[5], James P. Simmer[3], Jan C.-C. Hu[3]

[1]Department of Dentistry, National Taiwan University School of Dentistry, No.1, Changde St., Taipei City 100, Taiwan.
[2]Department of Pediatric Dentistry, National Taiwan University Children's Hospital, No.8, Zhongshan S. Rd., Taipei City 100, Taiwan.
[3]Department of Biologic and Materials Sciences, University of Michigan School of Dentistry, 1011 North University, Ann Arbor, MI 48108, USA.
[4]Department of Pediatric Dentistry, Faculty of Dentistry, Altinbas University, Istanbul, 34147, Turkey.
[5]Department of Pedodontics, Faculty of Dentistry, Istanbul University, Istanbul, 34116, Turkey.

Contents:

Figure S1. Intra-oral photographs and radiographs of Family 5 members

Table S1. *AMELX* disease-causing mutations

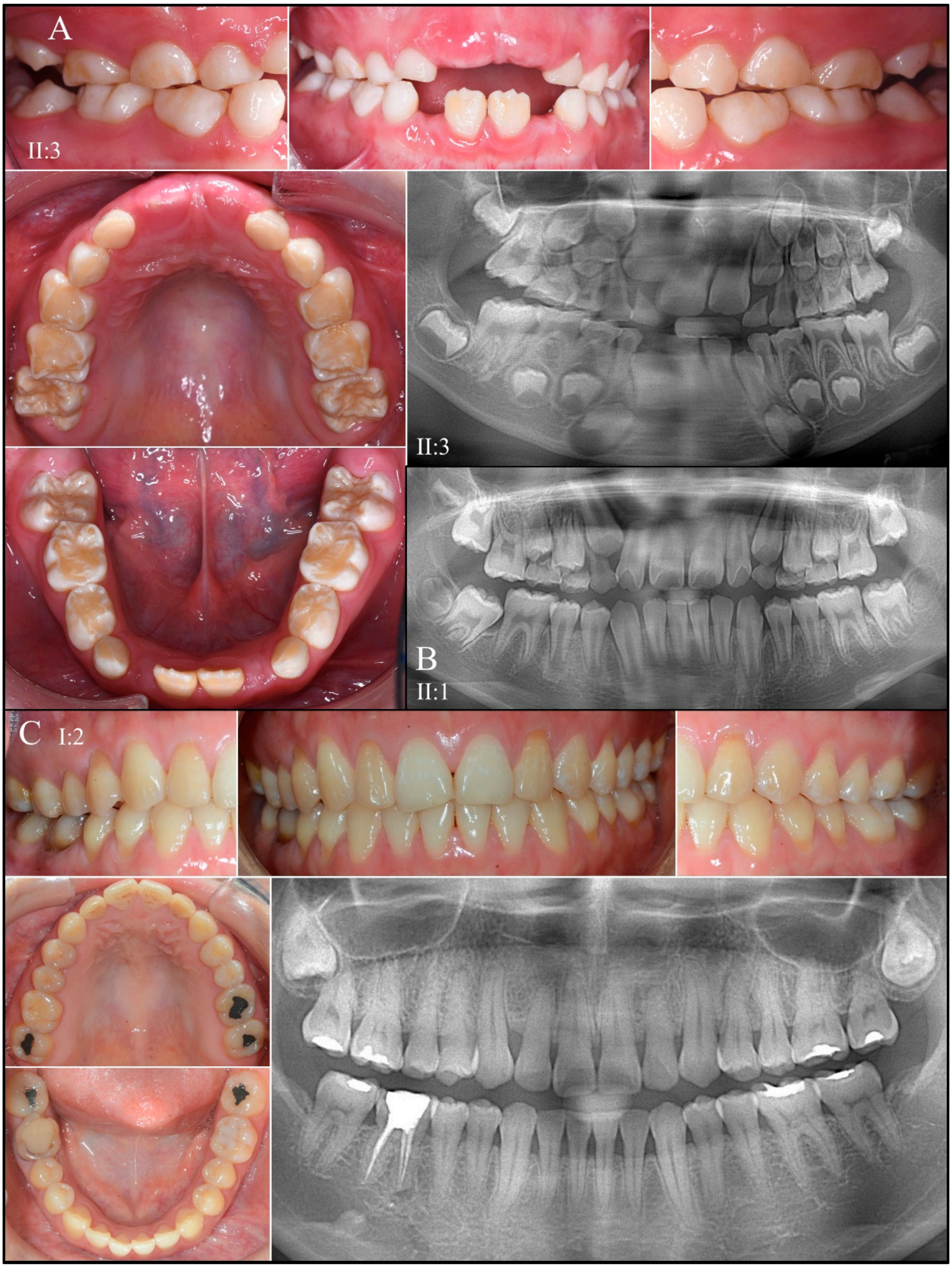

Figure S1. Intra-oral photographs and radiographs of Family 5 members. (A) Clinical photograph and radiograph of proband's younger brother (II:3) at age 8.5 shows enamel malformation similar to that of the proband, which was of reduced thickness and mineralization. (B) The panorex of proband's elder brother (II:1) at age 12 exhibits features of hypoplastic and hypomaturation AI, like his other two siblings. (C) The affected mother's (I:2, age 37) teeth appeared generally normal clinically, although hypomineralized white spots were evident on multiple teeth, particularly on the occlusal third of the buccal surfaces of the posterior teeth.

Table S1. *AMELX* disease-causing mutations

#	Exon	Gene (NG_012494.2)	cDNA (NM_182680.1)	Protein (NP_872621.1)	Ref
1		g.363924_416576del	c.-39354_*6167del	p.?	[1]
2		g.307534_403773del	c.-26548_*62560del	p.?	Family 5
3		g.302534_398773del	c.-21552_*67556del	p.?	[1]
4	2	g.375912A>G	c.2T>C	p.(Met1?)	Family 1, [2]
5	2	g.375903C>T	c.11G>A	p.(Trp4*)	[3]
6	2	g.375903C>G	c.11G>C	p.(Trp4Ser)	[2]
7	2	g.375892_375900del	c.14_22del	p.(Ile5_Ala8delinsThr)	[4]
8	2	g.375885A>G	c.29T>C	p.(Leu10Pro)	Family 2
9		g.375867G>T	c.47C>A	p.(Ala16Asp)	[3]
10		g.370045_374767del	c.55-840_*49del	p.?	[5]
11	3	g.373903del	c.77del	p.(Pro26Leufs*23)	Family 3
12		g.372602A>G	c.103-3A>C	p.?	[6]
13	4	g.372582A>G	c.120T>C	p.(Ala40=)	[7]
14	4	g.372559A>G	c.143T>C	p.(Leu48Ser)§	[8]
15		g.372467C>T	c.145-1G>A	p.?	Family 4
16	5	g.372459G>A	c.152C>T	p.(Thr51Ile)	[9]
17	5	g.372459del	c.155del	p.(Pro52Leufs*2)	[10-12]
18	5	g.372456G>C	c.155C>G	p.(Pro52Arg)	[13]
19	5	g.372456G>A	c.155C>T	p.(Pro52Leu)	[3,14]
20	5	g.372427del	c.185del	p.(Pro62Argfs*47)	[15]
21	6	g.372133G>T	c.208C>A	p.(Pro70Thr)	[3,12,16-19]
22	6	g.372111T>A	c.230A>T	p.(His77Leu)	[20]
23	6	g.372099G>A	c.242C>T	p.(Pro81Leu)	[21]
24	6	g.371957del	c.385del	p.(His129Thrfs*60)	[22]
25	6	g.371923del	c.420del	p.(Tyr141Thrfs*48)	[23]
26	6	g.371869del	c.473del	p.(Pro158Hisfs*31)	[3,9,12]
27	6	g.371824del	c.517del	p.(Pro173Leufs*16)	[24]
28	6	g.371803del	c.541del	p.(Leu181Cysfs*8)	[3,20,25]
29	6	g.371770C>A	c.571G>T	p.(Glu191*)	[9]
30		g.371728C>T	c.612+1G>A	p.?	[26]

References

1. Hu, J.C.; Chan, H.C.; Simmer, S.G.; Seymen, F.; Richardson, A.S.; Hu, Y.; Milkovich, R.N.; Estrella, N.M.; Yildirim, M.; Bayram, M.; et al. Amelogenesis imperfecta in two families with defined AMELX deletions in ARHGAP6. *PLoS One* **2012**, *7*, e52052, doi:10.1371/journal.pone.0052052.
2. Kim, J.W.; Simmer, J.P.; Hu, Y.Y.; Lin, B.P.; Boyd, C.; Wright, J.T.; Yamada, C.J.; Rayes, S.K.; Feigal, R.J.; Hu, J.C. Amelogenin p.M1T and p.W4S mutations underlying hypoplastic X-linked amelogenesis imperfecta. *J Dent Res* **2004**, *83*, 378-383, doi:10.1177/154405910408300505.
3. Bloch-Zupan, A.; Rey, T.; Jimenez-Armijo, A.; Kawczynski, M.; Kharouf, N.; Dure-Molla, M.; Noirrit, E.; Hernandez, M.; Joseph-Beaudin, C.; Lopez, S.; et al. Amelogenesis imperfecta: Next-generation sequencing sheds light on Witkop's classification. *Front Physiol* **2023**, *14*, 1130175, doi:10.3389/fphys.2023.1130175.
4. Lagerström-Fermér, M.; Nilsson, M.; Bäckman, B.; Salido, E.; Shapiro, L.; Pettersson, U.; Landegren, U. Amelogenin signal peptide mutation: correlation between mutations in the amelogenin gene (AMGX) and manifestations of X-linked amelogenesis imperfecta. *Genomics* **1995**, *26*, 159-162, doi:10.1016/0888-7543(95)80097-6.
5. Lagerström, M.; Dahl, N.; Nakahori, Y.; Nakagome, Y.; Bäckman, B.; Landegren, U.; Pettersson, U. A deletion in the amelogenin gene (AMG) causes X-linked amelogenesis imperfecta (AIH1). *Genomics* **1991**, *10*, 971-975, doi:10.1016/0888-7543(91)90187-j.
6. Leban, T.; Trebušak Podkrajšek, K.; Kovač, J.; Fidler, A.; Pavlič, A. An Intron c.103-3T>C Variant of the AMELX Gene Causes Combined Hypomineralized and Hypoplastic Type of Amelogenesis Imperfecta: Case Series and Review of the Literature. *Genes (Basel)* **2022**, *13*, doi:10.3390/genes13071272.
7. Cho, E.S.; Kim, K.J.; Lee, K.E.; Lee, E.J.; Yun, C.Y.; Lee, M.J.; Shin, T.J.; Hyun, H.K.; Kim, Y.J.; Lee, S.H.; et al. Alteration of conserved alternative splicing in AMELX causes enamel defects. *J Dent Res* **2014**, *93*, 980-987, doi:10.1177/0022034514547272.
8. Kim, Y.J.; Kang, J.; Seymen, F.; Koruyucu, M.; Zhang, H.; Kasimoglu, Y.; Bayram, M.; Tuna-Ince, E.B.; Bayrak, S.; Tuloglu, N.; et al. Alteration of Exon Definition Causes Amelogenesis Imperfecta. *J Dent Res* **2020**, *99*, 410-418, doi:10.1177/0022034520901708.
9. Lench, N.J.; Winter, G.B. Characterisation of molecular defects in X-linked amelogenesis imperfecta (AIH1). *Hum Mutat* **1995**, *5*, 251-259, doi:10.1002/humu.1380050310.
10. Aldred, M.J.; Crawford, P.J.; Roberts, E.; Thomas, N.S. Identification of a nonsense mutation in the amelogenin gene (AMELX) in a family with X-linked amelogenesis imperfecta (AIH1). *Hum Genet* **1992**, *90*, 413-416, doi:10.1007/bf00220469.
11. Lench, N.J.; Brook, A.H.; Winter, G.B. SSCP detection of a nonsense mutation in exon 5 of the amelogenin gene (AMGX) causing X-linked amelogenesis imperfecta (AIH1). *Hum Mol Genet* **1994**, *3*, 827-828, doi:10.1093/hmg/3.5.827.
12. Wright, J.T.; Torain, M.; Long, K.; Seow, K.; Crawford, P.; Aldred, M.J.; Hart, P.S.; Hart, T.C. Amelogenesis imperfecta: genotype-phenotype studies in 71 families. *Cells Tissues Organs* **2011**, *194*, 279-283, doi:10.1159/000324339.
13. Kida, M.; Sakiyama, Y.; Matsuda, A.; Takabayashi, S.; Ochi, H.; Sekiguchi, H.; Minamitake, S.; Ariga, T. A novel missense mutation (p.P52R) in amelogenin gene causing X-linked amelogenesis imperfecta. *J Dent Res* **2007**, *86*, 69-72, doi:10.1177/154405910708600111.
14. Prasad, M.K.; Geoffroy, V.; Vicaire, S.; Jost, B.; Dumas, M.; Le Gras, S.; Switala, M.; Gasse, B.; Laugel-Haushalter, V.; Paschaki, M.; et al. A targeted next-generation sequencing assay for the molecular diagnosis of genetic disorders with orodental involvement. *J Med Genet* **2016**, *53*, 98-110, doi:10.1136/jmedgenet-2015-103302.

15. Duan, X.; Yang, S.; Zhang, H.; Wu, J.; Zhang, Y.; Ji, D.; Tie, L.; Boerkoel, C.F. A Novel AMELX Mutation, Its Phenotypic Features, and Skewed X Inactivation. *J Dent Res* **2019**, *98*, 870-878, doi:10.1177/0022034519854973.
16. Chan, H.C.; Estrella, N.M.; Milkovich, R.N.; Kim, J.W.; Simmer, J.P.; Hu, J.C. Target gene analyses of 39 amelogenesis imperfecta kindreds. *Eur J Oral Sci* **2011**, *119 Suppl 1*, 311-323, doi:10.1111/j.1600-0722.2011.00857.x.
17. Collier, P.M.; Sauk, J.J.; Rosenbloom, S.J.; Yuan, Z.A.; Gibson, C.W. An amelogenin gene defect associated with human X-linked amelogenesis imperfecta. *Arch Oral Biol* **1997**, *42*, 235-242, doi:10.1016/s0003-9969(96)00099-4.
18. Hart, S.; Hart, T.; Gibson, C.; Wright, J.T. Mutational analysis of X-linked amelogenesis imperfecta in multiple families. *Arch Oral Biol* **2000**, *45*, 79-86, doi:10.1016/s0003-9969(99)00106-5.
19. Ravassipour, D.B.; Hart, P.S.; Hart, T.C.; Ritter, A.V.; Yamauchi, M.; Gibson, C.; Wright, J.T. Unique enamel phenotype associated with amelogenin gene (AMELX) codon 41 point mutation. *J Dent Res* **2000**, *79*, 1476-1481, doi:10.1177/00220345000790070801.
20. Hart, P.S.; Aldred, M.J.; Crawford, P.J.; Wright, N.J.; Hart, T.C.; Wright, J.T. Amelogenesis imperfecta phenotype-genotype correlations with two amelogenin gene mutations. *Arch Oral Biol* **2002**, *47*, 261-265, doi:10.1016/s0003-9969(02)00003-1.
21. Kim, Y.J.; Kim, Y.J.; Kang, J.; Shin, T.J.; Hyun, H.K.; Lee, S.H.; Lee, Z.H.; Kim, J.W. A novel AMELX mutation causes hypoplastic amelogenesis imperfecta. *Arch Oral Biol* **2017**, *76*, 61-65, doi:10.1016/j.archoralbio.2017.01.004.
22. Sekiguchi, H.; Alaluusua, S.; Minaguchi, K.; Yakushiji, M. A new mutation in the amelogenin gene causes X-linked amelogenesis imperfecta. *J Dent Res* **2001**, *80 Suppl*, 617.
23. Greene, S.R.; Yuan, Z.A.; Wright, J.T.; Amjad, H.; Abrams, W.R.; Buchanan, J.A.; Trachtenberg, D.I.; Gibson, C.W. A new frameshift mutation encoding a truncated amelogenin leads to X-linked amelogenesis imperfecta. *Arch Oral Biol* **2002**, *47*, 211-217, doi:10.1016/s0003-9969(01)00111-x.
24. Lee, K.E.; Lee, S.K.; Jung, S.E.; Song, S.J.; Cho, S.H.; Lee, Z.H.; Kim, J.W. A novel mutation in the AMELX gene and multiple crown resorptions. *Eur J Oral Sci* **2011**, *119 Suppl 1*, 324-328, doi:10.1111/j.1600-0722.2011.00858.x.
25. Kindelan, S.A.; Brook, A.H.; Gangemi, L.; Lench, N.; Wong, F.S.; Fearne, J.; Jackson, Z.; Foster, G.; Stringer, B.M. Detection of a novel mutation in X-linked amelogenesis imperfecta. *J Dent Res* **2000**, *79*, 1978-1982, doi:10.1177/00220345000790120901.
26. Zhang, Z.; Zou, X.; Feng, L.; Huang, Y.; Chen, F.; Sun, K.; Song, Y.; Lv, P.; Gao, X.; Dong, Y.; Tian, H. Splicing mutations in AMELX and ENAM cause amelogenesis imperfecta. *BMC Oral Health* **2023**, *23*, 893, doi:10.1186/s12903-023-03508-8.
